# Mutations in the mitochondrial DNA D-loop region are frequent in cervical cancer

**DOI:** 10.1186/1475-2867-5-34

**Published:** 2005-12-16

**Authors:** Himani Sharma, Archna Singh, Chandresh Sharma, Sunesh Kumar Jain, Neeta Singh

**Affiliations:** 1 Department of Biochemistry, All India Institute of Medical Sciences, New Delhi 110029, India; 2 Department of Obstetrics & Gynecology*, All India Institute of Medical Sciences, New Delhi 110029, India

## Abstract

**Background:**

Mitochondrial DNA (mtDNA) is known for high mutation rates caused by lack of protective histones, inefficient DNA repair systems, and continuous exposure to mutagenic effects of oxygen radicals. Alterations in the non-coding displacement (D) loop of mitochondrial DNA are present in many cancers. It has been suggested that the extent of mitochondrial DNA mutations might be useful in the prognosis of cancer outcome and/or the response to certain therapies. In order to investigate whether a high incidence of mutations exist in mitochondrial DNA of cervical cancer patients, we examined the frequency of mutations in the D-loop region in 19 patients of cervical cancer.

**Results:**

Mutations, often multiple, were detected in 18 of 19 (95%) patients. The presence of mutations correlated with Human Papilloma Virus (HPV) infection in these patients. Mutations were also detected in normal samples and lymphocytes obtained from cervical cancer patients, but their frequency of occurrence was much lower as compared to the cervical cancer tissues.

**Conclusion:**

Our findings indicate that D-loop alterations are frequent in cervical cancers and are possibly caused by HPV infection. There was no association of mtDNA D-loop mutations with the histopathological grade and tumor staging.

## Background

The human mtDNA is a double stranded circular molecule of 16569 bp and contains 37 genes coding for two rRNAs, 22 tRNAs and 13 polypeptides. It is present in high copy numbers (10^3^–10^4 ^copies per cell) in virtually all cells and the vast majority of copies are identical at birth. Furthermore, mtDNA is known for having high acquired mutation rates which are 10 times higher than that of nuclear genomic DNA. It is generally accepted that high mutation rates of mtDNA are caused by lack of protective histones, inefficient DNA repair systems and continuous exposure to mutagenic effects of oxygen radicals generated by oxidative phosphorylation [1]. An association between mtDNA mutations and neurologic or metabolic disorders has previously been reported [2-4].

The D-loop, which is 1124 bp in size (positions 16024-576), is a non-coding region, and acts as a promoter for both the heavy and light strands of the mtDNA, and contains essential transcription and replication elements. The D-loop region is a hot spot for mtDNA alterations, and it contains two hypervariable regions (HV1 at positions 16024–16383 and HV2 at positions 57–372). The sequence analysis of these two regions is used not only in forensic analyses, but also in medical diagnosis [5].

Mutations in the mtDNA have been reported to occur in human cancers [6-11]. Alonso et al [7] detected mutations in the mtDNA D-loop region in colorectal and gastric malignancies. There are other reports that have analyzed alterations of the D-loop in lung cancer, colon cancer, ovarian cancer, hepatocellular carcinoma and breast cancer [12-16].

Cervical cancer is a leading cause of cancer related death in females and is the second most common gynecologic malignancy worldwide [17]. Developing countries account for the majority (80%) of the 500,000 new cases diagnosed each year as well as for the significant proportion of the mortality (80%) [18]. Squamous cell carcinomas are the most common histologic type followed by adenocarcinomas and other rarer types in cervical cancer. The major biological risk factor implicated in the development of cervical cancer is the human papilloma virus (HPV). The causal role of human papillomavirus infections in cervical cancer has been verified by epidemiologic criteria, and HPV DNA has been shown to be present in over 91% of squamous cell carcinomas of the cervix [18].

In this study, we examined alterations of the D-loop region in cervical cancer tissues, and related our findings to clinicopathological features and HPV infection.

## Results and discussion

The purpose of this study was to evaluate the mutation frequencies in the two hypervariable regions (HV1 and HV2) and the region between the two hypervariable regions of the mtDNA D-loop in cervical cancer, and the correlation of these mutations with HPV infection. Nineteen patients with cervical cancer were included in the study.

While mitochondrial DNA mutations have been studied widely in the context of rare genetic diseases, their involvement in carcinogenesis has been relatively less studied [19]. Since mitochondria is the site of initiation of apoptosis, therefore, mutation of its genome may play a causative role in cancer. The reports that do exist on the subject appear inconsistent or at times contradictory e.g. Heerdt et al [13] found no mutations in the promoter for the H and L strands on the mtD-loop region, in a study done to determine a possible association with colon cancer. However, another report showed that 70% of the colon cancers examined displayed mitochondrial DNA mutations [20]. Among other tumor types studied, Tamura et al [8] analyzed the mutations of the mtDNA D-loop region including HV1 and HV2 in 45 Japanese patients with gastric cancer, and found mutations in 4% of the tumors. Conversely, Alonso et al [7] detected mutations in the mtDNA D-loop region in 23% colorectal tumors and 37% gastric tumors. Fliss et al [11] analyzed mutations in mtDNA in bladder, head and neck, and lung cancer and found mutations in 50% of cancers of which 67% were in the mtDNA D-loop region.

To the best of our knowledge, this is the first report to examine the involvement of mitochondrial mutations in cervical cancer. Except for one study [21], apparently, the involvement of mtDNA mutations in cervical cancer has not been examined. A search of literature failed to turn up any relevant studies. Hence, this study is a step in this direction. Mitochondrial mutations have been reported in other gynecological malignancies. In ovarian cancer 60% of somatic mutations in the D loop, 12 s rRNA, 16 s rRNA and cyt b have been reported [14]. Most of the mutations were T → C or G → A transitions. The only report on mtDNA mutations in cervical cancer has reported high frequency of D310 mutations in 35% of cases [21]. However, they have not looked at other mtDNA mutations besides D310.

We examined 19 cervical cancer tissues and found that 95% samples showed mutation within a 1 Kb region of the 16 Kb genome that we examined. If the same variation appeared in both cancer and normal tissue, it was considered as polymorphism. We also found mutations in the normal tissues and lymphocytes that we examined, but their frequency was lower than that observed in case of tumors. Most of the mutations in both normal and tumor DNA were single base substitutions along with insertions and deletions. We found novel mutations at 55 sites in case of tumors. Similar mutations were found in more than one sample. In case of controls, only 23 sites had these novel mutations whereas these mutations were present at only 9 sites in lymphocytes [Table 1]. Interestingly, the presence of mutations correlated significantly (p < 0.05) with HPV infection.

**Table 1:** Novel mutations detected in the mitochondrial D-loop region of cervical cancer tissues, controls and lymphocytes

	**Tumors**			**Controls**	
**Base pair**	**Mutation**	**No. of samples**	**Base pair**	**Mutation**	**No. of samples**

65	A insertion	1	5	T-C	1
105	C deletion	1	56	A-T	1
134	T-A	1	60	T deletion	1
144	C-T	1	303	A insertion	1
151	A insertion	1	311	A insertion	1
179	T-C	1	319	T insertion	1
224	T-C	1	323	C insertion	1
268	A insertion	1	336	A insertion	1
269	T insertion	1	337	A insertion	1
295	A insertion	1	343	T insertion	1
311	A insertion	1	371	C-A	1
317	C-T	1	375	A insertion	1
371	C-A	1	420	A insertion	1
378	C-T	1	427	A insertion	1
412	C-A	1	456	T insertion	1
424	T insertion	1	563	A-G	1
424	T-A	1	16184	T insertion	1
484	A-G	1	16189	T deletion	1
511	C-T	2	16318	A insertion	1
519	A-G	1	16333	A-T	1
563	A-G	1	16335	A insertion	1
16027	T-C	1	16356	A insertion	1
16045	T-A	1	16542	A insertion	1
16045	T insertion	1			
16064	T-A	1		**Lymphocytes**	
16068	T insertion	1	**Base pair**	**Mutation**	**No. of samples**
16068	T-A	1	29	A-C	1
16070	T insertion	1	61	A insertion	1
16073	C-A	1	65	T-A	1
16092	T deletion	1	379	A-C	1
16095	A insertion	1	16036	C insertion	1
16123	A insertion	1	16038	A-G	1
16137	A deletion	1	16065	G-A	1
16143	T deletion	1	16067	A insertion	1
16157	T-A	1	16109	T insertion	1
16170	A deletion	1			
16177	A-G	1			
16180	A deletion	2			
16212	A insertion	2			
16214	A insertion	2			
16215	T deletion	2			
16220	A deletion	1			
16222	C deletion	1			
16225	C-T	1			
16289	T insertion	1			
16293	A deletion	1			
16367	A deletion	1			
16381	C insertion	2			
16383	T insertion	1			
16396	C insertion	2			
16402	C insertion	1			
16405	C insertion	1			
16406	T insertion	1			
16408	C insertion	1			
16413	T insertion	1			
16414	T insertion	1			
16420	A deletion	1			
16428	C insertion	1			
16434	A insertion	1			

Furthermore, we noted a high rate of polymorphism in the DNA obtained from both normal and tumor tissue. We found polymorphisms at 81 sites in tumors, at 26 sites in controls and at 13 sites in lymphocytes.

A high frequency of mitochondrial microsatellite instability (mtMSI) was detected in the D-loop region in these samples. We observed mtMSI in two different regions, including CA repeats of nucleotide positions 514–523 within a nonfunctional D-loop sequence (1/19) and a common homopolymeric C stretch interrupted by a T (CCCCCTCCCC) at nucleotide positions 303–315 (11/19). The mtMSI within the mononucleotide repeat at nucleotide position 303–309 has been frequently observed in endometrial tumors [21]. This mtMSI has also been detected in ovarian carcinomas [14]. This region is part of the binding site of replication primer. Variation in the CA repeats was detected in only one of the cervical carcinoma samples. Instability of the CA repeats has also been seen at a low frequency in endometrial [22], ovarian [14], gastric cancers [23] and glioblastoma [24]. However, there is a significantly higher frequency of the CA-repeat instability in breast cancer (42.5%) [25]. On the basis of these findings, we suggest that somatic mtMSI occurs differentially in different tumors.

Malfunctions of mismatch repair genes have been associated with nuclear MSI. However, to date, no mismatch repair genes have been found responsible for the maintenance of mammalian mitochondrial genome. The occurrence of high frequency of mtMSI in cervical cancers indicates that DNA repair in mitochondria is highly inefficient after malignant transformation with HPV. The different frequencies of occurrence of the mtMSI at the mononucleotide repeat at nucleotide position 303–315 in different tumor cell types suggests that deficits in mismatch repair function in mitochondria may vary from one tumor to another.

The occurrence of mtMSI may derive mainly from erroneous replication, whereas point mutations may be attributed to oxidative damage caused by reactive oxygen species. In addition, a study [26] suggests that homopolymorphic nucleotide tracts in mtDNA are error prone because of the low frameshift fidelity of the DNA polymerase gamma that carries out the mtDNA replication. Thus, mtMSI in cervical cancers may be mainly attributed to errors of replication. Moreover, there is supportive evidence that there are cell-specific differences in the repair of mtDNA damage [27]. The differential occurrence of mtDNA mutations in various cancers may be partly due to the presence of different repair mechanisms in different tumor cell types.

We also found novel polymorphisms at 11 sites in tumors and at 4 sites in normal tissue [Table 2]. There was a substantial difference in the frequency of mutations between patients in our study. The mutations detected ranged from 1 to 13 within the 1 kb region that we examined. We were unable to draw any correlations between the number of mutations and clinical characteristics.

**Table 2:** Novel polymorphisms detected in the mitochondrial D-loop of cervical cancer tissues and controls

	**Tumors**			**Controls**	
**Base pair**	**Polymorphism**	**No. of samples**	**Base pair**	**Polymorphism**	**No. of samples**

113	C-A	1	57	T-A	1
141	A insertion	2	113	C-A	1
268	C-A	1	268	C-A	1
316	G-C	1	295	C-A	1
16124	T-A	2			
16126	T-G	1			
16162	A-T	1			
16163	A-C	1			
16178	T-G	1			
16180	A-T	1			
16342	T-A	1			

## Conclusion

The study indicates that mitochondrial mutations are indeed frequent in cervical cancer cells and further work is warranted in this direction. Further, given the critical role of mitochondria in apoptosis, perhaps the mutations in mtDNA in cancer cells could significantly affect the cellular apoptotic response to chemotherapeutic agents. However, we did not find any correlation between mtDNA mutation and apoptotic index or apoptosis related pro- and anti-apoptotic proteins (data not shown).

## Methods

### Sample collection and DNA extraction

A total of 24 surgically resected cervical tissue samples obtained after informed consent from the Department of Obstetrics & Gynecology, All India Institute of Medical Sciences, New Delhi, India were collected and frozen at -70°C. The age of the patients ranged from 29 yrs to 65 yrs [Table 3]. None of them had received any prior radiation therapy or chemotherapy. These samples consisted of 17 squamous cell carcinoma 1 adeno-squamons and 1 adenocarcinoma, of the cervix, and 5 histopathologically normal cervical tissue obtained from women undergoing hysterectomy for reasons other than cervical cancer [Table 3]. Also, representative sections of cervical tissue were stained with eosin and hematoxylin for confirmation of diagnosis. The tumor samples contained at least 70% of malignant cells. Lymphocytes were also obtained from cervical cancer patients whose tumor tissues were collected.

**Table 3:** Clinicopathological features of patients included in the study, and presence of HPV infection.

		**Tumors**			
**Case No.**	**Age (Years)**	**Stage**	**H/P type**	**Grade**	**HPV type**

3	65	IIb	LCNK	MD	HPV-16
5	60	Ia	LCNK	MD	neg
6	29	Ia	LCNK	MD	HPV-16
12	40	Ib	LCNK	MD	HPV-16
17	35	IIb	LCNK	MD	HPV-16
18	50	Ia	LCNK	MD	HPV-16
23	43	Ia	KSCC	MD	HPV-16
24	42	IIIb	LCNK	MD	HPV-16
28	48	IIb	LCNK	MD	HPV-16
33	40	IIIb	LCNK	MD	HPV-16
36	65	IIb	LCNK	PD	HPV-16
38	35	IIb	LCNK	MD	HPV-16
42	35	IIb	LCNK	PD	HPV-16
50	38	IIa	AS	MD	HPV-16
53	45	Ia	LCNK	MD	HPV-16
56	35	Ia	LCNK	MD	neg
60	35	Ib	LCNK	MD	HPV-16
61	42	Ib	AD	MD	HPV-16
62	47	Ib	LCNK	MD	HPV-16
		**Controls**			
1	42	NA	NA	NA	neg
2	55	NA	NA	NA	neg
3	40	NA	NA	NA	neg
4	50	NA	NA	NA	neg
5	42	NA	NA	NA	neg

H/P-Histopathological; NA-Not applicable; neg-negative; ND-Not determined;

LCNK-large cell non-keratizing; AS-Adenosquamous carcinoma; AD-Adenocarcinoma;

MD-Moderately differentiated; PD-Poorly differentiated.

### DNA isolation and analysis

Total DNA (nuclear and mitochondrial) was isolated as described earlier [18]. The samples were digested with Proteinase K, extracted with phenol-chloroform, and ethanol precipitated. The quality of the DNA was checked by PCR for β globin as an internal control. This DNA was used to amplify the entire D-loop region of the mitochondrial genome known to have high mutation rates. The region which spans sequences from 15964–16439 was amplified using the forward and reverse primers as follows: 5'-AAAGTCTTTAACTCCACC-3' and 5'-GCACTCTTGTGCGGGATATTG-3'. The region from 16531-740 was amplified with the following primers 5'-AATAGCCCACACGTTCCCCT-3' and 5'-CGTGGTGATTTAGAGGGTGA-3'. The two fragments thus obtained were cloned into the TA cloning vector pGEM-TEasy (Promega, USA) and were subjected to sequencing using the M13 forward and reverse primers. Sequences were compared against a mitochondrial data base [28]. Nuclease-free water was used as a negative control for PCR instead of DNA.

HPV testing was done as described earlier [18] using consensus primers MY09/11 from the L1 region. The primer sequences for HPV 16 primers were 5' CCC AGC TGT AAT CAT GCA TGG AGA 3' and 5' GTG TGC CCA TTA ACA GGT CTT CCA 3' and HPV-18 primers were 5'AAGGATGCTGCACCGGCTGA 3' and 5' CACGCACACGCTTGGCAGGT 3'.

### Statistical analysis

Correlation of presence of mitochondrial DNA mutations with HPV infection was done using the chi-square test. A p value < 0.05 was considered statistically significant.

## Authors' contributions

HS carried out the experimental work, did BLAST searches, analyzed data and drafted the manuscript. AS collected the samples for the study and performed DNA extraction. CS participated in the cloning of PCR products in pGEMTEasy vector. SKJ provided the samples for the study. NS designed the study and helped in drafting the manuscript. All authors read and approved the final manuscript.
